# Genotype-by-socioeconomic status interaction influences heart disease risk scores and carotid artery thickness in Mexican Americans: the predominant role of education in comparison to household income and socioeconomic index

**DOI:** 10.3389/fgene.2023.1132110

**Published:** 2023-09-14

**Authors:** Vincent P. Diego, Eron G. Manusov, Xi Mao, Joanne E. Curran, Harald Göring, Marcio Almeida, Michael C. Mahaney, Juan M. Peralta, John Blangero, Sarah Williams-Blangero

**Affiliations:** ^1^ Department of Human Genetics, School of Medicine, University of Texas Rio Grande Valley, Brownsville, TX, United States; ^2^ School of Medicine, South Texas Diabetes and Obesity Institute, University of Texas Rio Grande Valley, Brownsville, TX, United States; ^3^ Department of Economics, University of Texas Rio Grande Valley, Brownsville, TX, United States

**Keywords:** GxE, CVD (cardio vascular disease), Duncan’s SEI, SES, Mexican Americans, Framingham 10-year general cardiovascular disease risk, linear mixed model

## Abstract

**Background:** Socioeconomic status (SES) is a potent environmental determinant of health. To our knowledge, no assessment of genotype-environment interaction has been conducted to consider the joint effects of socioeconomic status and genetics on risk for cardiovascular disease (CVD). We analyzed Mexican American Family Studies (MAFS) data to evaluate the hypothesis that genotype-by-environment interaction (GxE) is an important determinant of variation in CVD risk factors.

**Methods:** We employed a linear mixed model to investigate GxE in Mexican American extended families. We studied two proxies for CVD [Pooled Cohort Equation Risk Scores/Framingham Risk Scores (FRS/PCRS) and carotid artery intima-media thickness (CA-IMT)] in relation to socioeconomic status as determined by Duncan’s Socioeconomic Index (SEI), years of education, and household income.

**Results:** We calculated heritability for FRS/PCRS and carotid artery intima-media thickness. There was evidence of GxE due to additive genetic variance heterogeneity and genetic correlation for FRS, PCRS, and CA-IMT measures for education (environment) but not for household income or SEI.

**Conclusion:** The genetic effects underlying CVD are dynamically modulated at the lower end of the SES spectrum. There is a significant change in the genetic architecture underlying the major components of CVD in response to changes in education.

## Introduction

Cardiovascular disease (Tsao et al., 2022) (CVD) is a significant cause of mortality globally (Barquera et al., 2015; Joseph et al., 2017; Roth et al., 2020) and is the leading cause of mortality in the United States (US) (Ahmad and Anderson, 2021; Murphy et al., 2021; Tsao et al., 2022). The stall and even decline in US life expectancy since 2010 is primarily due to CVD mortality (Roth et al., 2018; Mehta et al., 2020). A study of high-income countries, including Austria, Belgium, Germany, Italy, Netherlands, Sweden, Switzerland, and the United Kingdom, similarly implicated CVD mortality as a leading cause of life expectancy decline (Ho and Hendi, 2018).

Results from the Whitehall I Study, conducted over 4 decades ago, and the Whitehall II study, conducted 2 decades later, demonstrate a social gradient describing an inverse relationship between mortality (particularly CVD mortality) and morbidity, and socioeconomic status (SES) (Mar et al., 1978; Rose and Marmot, 1981; Marmot, 1982; Marmot and Wilkinson, 1986; Marmot, 1989) The pattern of an inverse relationship between mortality and morbidity outcomes and socioeconomic status (SES) determinants has since been substantiated across numerous studies. (Marmot et al., 1991; Marmot and Bartley, 2002; Marmot, 2003; de Mestral and Stringhini, 2017; Tillmann et al., 2017; Schultz et al., 2018; Carter et al., 2019; Rosengren et al., 2019; Yusuf et al., 2020; Powell-Wiley et al., 2022). The disparities in mortality and obesity-related health issues by SES are increasing, worsening the social gradient (Bosworth, 2018) particularly prevalent in Mexican Americans (Hazuda et al., 1988; Hazuda et al., 1991).

We conducted a statistical genetic investigation of the potential role of genotype-by-environment interaction (GxE) in mediating the social gradient related to CVD. SES is a composite environment that dynamically modulates the genetic architecture underlying CVD mortality and morbidity phenotypes. Our approach tests predictions derived from the general hypothesis that the complex genotype-phenotype map governing CVD outcomes is dependent upon or a function of the SES environment. Using three measures of SES [household income (INC), education years (EDU), and Duncan’s socioeconomic index (SEI)], we assessed the evidence for genotype by socioeconomic status in the Mexican American Family Studies (MAFS). We investigate potential genotype-by-SES interaction (GSI) influencing several atherosclerosis or CVD risk measures. Since there are no specific risk-scores for Mexican Americans, we used the established Framingham Risk Score (FRS-08), which was developed initially as a 10-year atherosclerosis risk assessment (Wilson et al., 1998) and later revised in 2008 as a more general CVD risk prediction algorithm; (D'Agostino et al., 2008); and the American College of Cardiology/American Heart Association (ACC/AHA) atherosclerotic CVD risk score, (Goff et al., 2014), which is a 10-year risk of atherosclerosis score (Pooled Cohort Equation Risk Score) developed for African Americans (PCE-AA) and Caucasian Americans (PCE-CA) (Boateng et al., 2018; Topel et al., 2018; Wang et al., 2018; Rospleszcz et al., 2019; Ko et al., 2020; Wekesah et al., 2020). Our measures of CVD risk (based on research published by our research team) include quantitative measures of carotid intima-media thickness (IMT), including the common carotid artery IMT (CCAIMT), common carotid artery far-wall IMT (CCAFIMT), internal carotid artery IMT (ICAIMT), and internal carotid artery far-wall IMT (ICAFIMT). (Tsao et al., 2022; Centurión, 2016; Polak and O’Leary, 2016; Melton et al., 2013).

We propose that SES acts at the cellular and molecular levels to modify gene expression regarding the risk of CVD and that this can be captured by GSI modeling.

## Materials and methods

The Institutional Review Board at the University of Texas Health Science Center at San Antonio approved the MAFS study protocols. All study participants provided written informed consent.

### Study population

As described by our team in earlier publications, the MAFS population (1431) comprises large Mexican American extended families (42) randomly ascertained with respect to CVD or comorbidities (Hazuda et al., 1988; Hazuda et al., 1991; Mitchell et al., 1996; MacCluer et al., 1999). The probands were recruited from a single census tract of a low-income San Antonio, Texas neighborhood. Inclusion criteria included the proband age between 40 years and 60 years, a spouse willing to participate in the study, and at least six relatives [first-, second-, and third-degree relatives of the proband (≥16 years) and of the probands spouse] available to participate in the study. Surveys determined social, behavioral, and lifestyle factors (past medical history, educational background, household income level, reproductive history, and smoking and alcohol use) related to cardiovascular risk. Established questionnaires accessed physical activity and food frequency (physical activity measured in units of metabolic-equivalent-tasks (METs), Stanford 7-Day Physical Activity Recall Instrument, and data on dietary intake variables (food frequency questionnaire developed specifically for the Mexican American population of San Antonio) (Hazuda et al., 1991).

### Phenotypic assessments

Traditional MS risk factors [fasting glucose (mg/d), fasting insulin (u/mL), 2-h glucose (mg/dL), leptin (ng/mL), high-density lipoprotein-cholesterol (mmol/L), triglycerides (mmol/L), total serum cholesterol (mmol/L), 2-h insulin (u/mL), body mass index (kg/m^2^), waist circumference (cm), systolic blood pressure (mmHg), diastolic blood pressure (mmHg) were collected on all participants (Mitchell et al., 1996; MacCluer et al., 1999). We excluded 291 individuals with diabetes, diabetes medications, hypertension, or dyslipidemia to account for the impact of altered metabolism due to disease or medication (Final sample size = 1140 individuals).

### Interview data

We collected DM status, DM medications, HTN medications, dyslipidemia medications, smoking history, leisure time, physical activity-work, total physical activity, diet intake-protein (g/d), carb (g/d), saturated fat (g/d), monosaturated fat (g/d), polyunsaturated fat (g/d), cholesterol (g/d), sucrose (g/d), alcohol (g/d)]. Our SES variables obtained at interview were INC, and EDU where SEI was computed following the method in (Hazuda et al., 1988; Hazuda et al., 1991). This variable was used as our index of the SES environment in all our GxE analyses.

### Heart disease risk and carotid artery intima-media thickness

The 10-year atherosclerosis scores were computed by applying the appropriate formulas to the requisite MAFS data for the FRS, PCE-AA, and PCE-CA (D’Agostino et al., 2008; Goff et al., 2014). The PCE scores were also race-specific. All three risk scores used age, total cholesterol (TC), high-density lipoprotein-cholesterol (HDL-C), systolic blood pressure (SBP), diabetes status, and smoking status as predictors. The PCE algorithms variably modeled interactions (depending on their statistical significance) between age and the other main predictors, whereas the FRS algorithm did not. The protocol for measuring the intima-media thicknesses is reported by Melton et al. (2013).

## Statistical analysis

### The polygenic model

Our statistical genetic approach is a linear mixed model, where for a generic phenotype vector **y** we write:

y=Xβ+g+e,

where X is a matrix of covariates augmented at the left by a column of 1s, β is a vector of the intercept parameter and corresponding regression coefficients, and g and e are unobserved random genetic and environmental effects, respectively. (Blangero et al., 2013; Diego et al., 2015a) The phenotypic covariance matrix, denoted by Σ , is given as:

$$\Sigma =K\sigma_{g}^{2}+I\sigma_{e}^{2},$$

where K and I respectively, give genetic relationship and identity matrices, and $\sigma_{g}^{2}$ and $\sigma_{e}^{2}$ are correspondingly the additive genetic and environmental variance components. We report the heritability ( $h^{2}$ ) of each trait under this “polygenic” model, defined as the ratio of the additive genetic variance to the total phenotypic variance, $h^{2}=\frac{\sigma_{g}^{2}}{\sigma_{g}^{2}+\sigma_{e}^{2}}=\frac{\sigma_{g}^{2}}{\sigma_{p}^{2}}$ . This is a coarse measure of the extent of genetic effects underlying a trait (Blangero et al., 2013; Diego et al., 2015a). Each phenotype was regressed against age, sex, age-squared, sex-by-age, and sex-by-age-squared, and then the regression residuals derived for each trait were normalized using a normal inverse transformation (Blangero et al., 2013). There were no sex-specific effects following the genotype-by-sex-interaction model (Diego et al., 2015b).

### Modeling genotype-by-environment interaction for continuous environments

The polygenic model is used to obtain estimates of trait heritabilities and as a model reference point upon which more complex models can be elaborated. For a sample of related individuals, assuming fully uncorrelated genetic and environmental effects, the polygenic model posits that the phenotypic covariance is decomposable into additive genetic and residual environmental variance components and that inter-individual covariances will be given strictly by the additive genetic variance weighted by the genetic relatedness coefficient. The latter feature of the polygenic model makes two implicit assumptions regarding the genetic covariance: that the pairwise genetic correlation is unity, and that the additive genetic variance is homogeneous.

Under the general GxE model, we relax these assumptions by expressing both the additive genetic variance and genetic correlations as continuous functions of a specific environment to capture any potential interaction between the genetic effects (i.e., the additive genetic variance and/or genetic correlation) and the specific environment. The null hypothesis under this extension is that the expression of the aggregate of all genotypes underlying a phenotype (polygenotype) is independent of the specific environment. Rejection of this null implies that the genotype-phenotype map for the trait in question depends on a specific environment or, in other words, is a function of the specific environment. We propose that the extent to which the genotype-phenotype interaction depends on a function of the environment can be captured by modeling the GxE variance. For the simplest case of contrasting two different SES environments, for example, high versus low levels of income, the GxE variance is zero if the following two conditions are simultaneously true: homogeneity in the additive genetic variance: $\sigma_{g1}^{2}=\sigma_{g2}^{2}=\sigma_{g}^{2}$ , where $\sigma_{g1}^{2}$ and $\sigma_{g2}^{2}$ are the additive genetic variances in environments 1 and 2 (low and high-income levels in the current example), respectively; the genetic correlation ( $\rho_{g}$ ) is one across environments: $\rho_{g}=1$ . Denoting the GxE variance as $\sigma_{g\Delta }^{2}$ , we have the expression:

$$\sigma_{g\Delta }^{2}=\left\{\begin{array}{l} \sigma_{g1}^{2}+\sigma_{g2}^{2}-\rho_{g}\sigma_{g1}\sigma_{g2}\forall \sigma_{g1}^{2}\neq \sigma_{g2}^{2}\\ 2\sigma_{g}^{2}\left(1-\rho_{g}\right)\forall \sigma_{g1}^{2}=\sigma_{g2}^{2}=\sigma_{g}^{2} \end{array}\right.$$

There is GxE evidence if either null hypothesis is rejected (Diego et al., 2003; Diego et al., 2007; Santos et al., 2014; Arya et al., 2018; Manusov et al., 2022). Rejection of either or both is evidence that the phenotypic response to the environment has a genetic basis.

We now extend this theory to a spectrum of such measures to model GxE for continuous SES environments as opposed to two levels of the SES variable. To this end, we employ variance and correlation functions (Diego et al., 2003; Diego et al., 2007; Santos et al., 2014; Arya et al., 2018; Manusov et al., 2022), which we now define as:

$$\sigma_{g}^{2}=\exp\left[\alpha_{g}+\gamma_{g}\left(q_{i}-\bar{q}\right)\right],\text{and }\rho_{g}=\exp\left(-\lambda_{g}\left|q_{i}-q_{j}\right|\right),$$

where the additive genetic variance is re-parameterized as an exponential function of the value of the environmental variable q for the ith individual, $q_{i}$ , scaled against the sample mean, $\bar{q}$ , and where the genetic correlation is re-parameterized as an exponential decay function of the difference of environmental variables for any pair of individuals i and j , and where $\alpha_{g}$ , $\gamma_{g}$ , and $\lambda_{g}$ are parameters to be estimated. These functions can be interpreted as the variance and correlation functions of a Gaussian stationary stochastic process, (Kirkpatrick and Heckman, 1989; Pletcher and Geyer, 1999; Jaffrézic and Pletcher, 2000; Pletcher and Jaffrézic, 2002; Diego et al., 2003; Meyer and Kirkpatrick, 2005) where the index variable of the process is one of the three quantitative SES variables. The statistical null hypotheses under the re-parameterizations is defined by variance homogeneity and genetic correlation stationarity at unity, respectively, is for $\gamma_{g}=0$ and $\lambda_{g}=0$ . To guard against model misspecification bias, we also model the residual environmental variance as a function of the environment in the same way as the additive genetic variance. We now define a genetic covariance matrix $\Psi =\left\{\psi_{ij}\right\}$ , with elements given by the variance and covariance functions of a Gaussian stationary stochastic process:

$$\psi_{ij}=\left\{\begin{array}{l} \sigma_{g}^{2}\forall i=j;\;q_{i}=q_{j}\\ \sigma_{i}{\sigma_{j}\rho }_{g}\forall i\neq j;\;q_{i}\neq q_{j} \end{array}\right.=\left\{\begin{array}{l} \exp\left[\alpha_{g}+\gamma_{g}\left(q_{i}-\bar{q}\right)\right]\forall i=j;\;q_{i}=q_{j}\\ {\left\{\exp\left[\alpha_{g}+\gamma_{g}\left(q_{i}-\bar{q}\right)\right]\right\}}^{\frac{1}{2}}\;{\left\{\exp\left[\alpha_{g}+\gamma_{g}\left(q_{j}-\bar{q}\right)\right]\right\}}^{\frac{1}{2}}\\ \exp\left(-\lambda_{g}\left|q_{i}-q_{j}\right|\right)\forall i\neq j;\;q_{i}\neq q_{j}. \end{array}\right.=\left\{\begin{array}{l} \exp\left[\alpha_{g}+\gamma_{g}\left(q_{i}-\bar{q}\right)\right]\forall i=j;\;q_{i}=q_{j}\\ \exp\left[\alpha_{g}+{\frac{1}{2}\gamma }_{g}\left(q_{i}+q_{j}-2\bar{q}\right)-\lambda_{g}\left|q_{i}-q_{j}\right|\right]\forall i\neq j;\;q_{i}\neq q_{j}. \end{array}\right.$$

We then posit a diagonal matrix $\Delta =\mathrm{diag}\left\{\delta_{ii}\right\}$ with diagonal elements containing the residual environmental variance function, $\delta_{ii}=\exp\left[\alpha_{e}+\gamma_{e}\left(q_{i}-\bar{q}\right)\right]$ . The covariance matrix for this GxE model for continuous environments is now given as follows:

Σ=K⊙Ψ+Δ,

where ⊙ denotes the Hadamard matrix multiplication operator. The continuous environment (i.e., the index of the Gaussian stationary stochastic process) analyzed under this model is INC, EDU, or SEI taken one at a time.

To ensure that we are highlighting the potential for SES environments to modulate the genotype-phenotype map, we employ Best Linear Unbiased Prediction (BLUP) methods to extract any associated additive genetic effects influencing EDU, INC, and SEI. (Quillen et al., 2014; Diego et al., 2015a; Porto et al., 2018). BLUP accounts for additive genetic and environmental covariances among relatives based on known pedigree structure (Diego et al., 2015a). We then subtracted the BLUP genetic values from the original SES variable to get a BLUP-computed version of that variable that reflects primarily environmental effects (Diego et al., 2015a; Manusov et al., 2022). This lattermost variable is our GXE models’ focal (genetically corrected) environment.

### Statistical inferential theory

For the basic polygenic model, denote the parameter vector by $\theta ={\left[\beta ,\sigma_{g}^{2},\sigma_{s}^{2},\sigma_{e}^{2}\right]}^{\prime }$ and the residuals vector by $r=\left(y-X\beta \right)$ . On assuming multivariate normality of the phenotype vector, the log-likelihood function is:

$$lnL\left(\theta |y,X\right)=\frac{-1}{2}\left[Nln2\pi +\ln\left|\Sigma \right|+r{\prime \Sigma }^{-1}r\right].$$

The statistical genetics package SOLAR (http://solar-eclipse-genetics.org/brief-overview.html) was used to obtain the model likelihoods, maximum likelihood estimates (MLEs) of model parameters, and their standard errors (SEs) (Almasy and Blangero, 1998). Hypothesis tests were performed by way of the likelihood ratio test (LRT) statistic, denoted as Λ :

$$\Lambda =-2\left[lnL\left(\theta_{N}\right)-lnL\left(\hat{\theta }\right)\right],$$

where $\theta_{N}$ denotes the parameter vector where a single parameter of interest is constrained to 0, all other parameters are unconstrained or free to be estimated at their MLEs for that model, and $\hat{\theta }$ denotes the fully unconstrained parameter vector where all parameters are estimated at their MLEs. As detailed above, this likelihood-based inferential procedure is extended to the GEI model by specifying the covariance matrix, Σ , and modifying the parameter vector, θ .

We adopted a two-stage hypothesis testing approach that our team has implemented to analyze GxE models of biomedically significant trait-environment dyads (Santos et al., 2014; Arya et al., 2018; Manusov et al., 2022). In the first stage, we examined if the overall GxE model provided a better fit to the data when compared with the polygenic model by the LRT. It is important to note that the polygenic model is nested within the full GxE model and that relative to the polygenic model, the GxE model has three additional parameters ( $\gamma_{g}$ , $\gamma_{e}$ , and $\lambda_{g}$ ; where $\alpha_{g}$ and $\alpha_{e}$ are re-parameterized versions of the variances). The LRT statistic for this comparison is distributed as a 50:50 mixture of chi-squares with 2 and 3 degrees of freedom (df) (Diego et al., 2003; Diego et al., 2007; Santos et al., 2014; Arya et al., 2018; Manusov et al., 2022).

In the second stage, we examined the more specific GxE hypotheses. The full GxE model with all parameters estimated was compared with models when either $\gamma_{g}$ or $\lambda_{g}$ was constrained to 0 to test the hypotheses of additive genetic variance homogeneity and a genetic correlation equal to one. The distributions of the LRT statistics are a chi-square with 1 df and a 50:50 mixture of a chi-square with a point mass at 0 and a chi-square with 1 df. (Diego et al., 2003; Diego et al., 2007; Santos et al., 2014; Arya et al., 2018; Manusov et al., 2022) Regarding the residual environmental variance function, $\gamma_{e}$ was constrained to 0 to test for homogeneity, where the LRT for this test is distributed as a chi-square with 1 df. As part of this stage, we determined if each of the three additional parameters in the full GEI model ( $\gamma_{g}$ , $\gamma_{e}$ , and $\lambda_{g}$ ) should be included by comparing its MLE to its SE A parameter is likely significant if its MLE is greater than twice its SE based on likelihood theory (Kirkpatrick and Heckman, 1989; Almasy and Blangero, 1998; Pletcher and Geyer, 1999; Jaffrézic and Pletcher, 2000; Pawitan, 2001; Pletcher and Jaffrézic, 2002; Meyer and Kirkpatrick, 2005; Quillen et al., 2014; Porto et al., 2018). Therefore, if a parameter SE was greater than its MLE, we judged that parameter to be statistically unimportant. Further, the additional parameters were formally tested by the tests mentioned above. If any of the three additional parameters were found to have SEs greater than their MLEs and if these were found to be formally insignificant, we then used a reduced version of the GxE model, where the parameter judged to be unimportant and found formally to be non-significant was constrained to 0, to perform the more specific hypothesis tests of the remaining parameters of interest.

## Results

### Genetic factors and socioeconomic measures

Our analysis reveals that genetic factors play a significant role in shaping the phenotypic response to various socioeconomic (SES) measures. The heritability estimates indicate that genetic factors account for a substantial portion of the variation in these traits, ranging from 41% to 51%. Table 1 provides an overview of the heritabilities and demographic data (EDU-h2 = 0.41, INC-h2 = 0.43, and SEI-H2 = 0.51 *p* < 1.0 s 10–5 for each).

**TABLE 1:** Descriptive statistics and heritabilities of variables.

Variables	Total		Males		Females		h2 (SE.)
	Mean (SD.)	N	Mean (SD.)	N	Mean (SD.)	N	
Environments (before BLUP)							
EDU	10.16 (3.92)	1232	10.26 (4.82)	506	10.08 (3.14)	726	0.41 (0.04)
INC	9.87 (3.01)	1067	9.95 (3.08)	418	9.82 (2.98)	649	0.43 (0.07)
SEI	29.73 (14.41)	1195	29.25 (14.42)	493	30.08 (14.4)	702	0.51 (0.06)
Risk Scores							
FRS 08	0.096 (0.147)	1241	0.097 (0.148)	506	0.094 (0.146)	735	0.28 (0.05)
PCE-AA	0.176 (0.274)	1241	0.184 (0.283)	506	0.171 (0.268)	735	0.23 (0.06)
PCE-CA	0.143 (0.22)	1241	0.152 (0.228)	506	0.137 (0.214)	735	0.17 (0.05)
Carotid Intima Media Thickness Measures							
CCAIMT	0.68 (0.16)	681	0.7 (0.19)	263	0.66 (0.14)	418	0.28 (0.08)
CCAFIMT	0.67 (0.19)	677	0.71 (0.23)	260	0.65 (0.16)	417	0.24 (0.08)
ICAIMT	0.83 (0.4)	657	0.88 (0.46)	256	0.8 (0.35)	401	0.32 (0.07)
ICAFIMT	0.83 (0.42)	610	0.89 (0.5)	243	0.79 (0.35)	367	0.21 (0.08)
Other							
Age (years)	37.59 (16.24)	1292	37.14 (16.88)	528	37.89 (15.79)	764	NA

Education is measured in years. Income is separated in >5 categories and therefore treated as continuous. SEI, is an index.

### Comparison of genotype-by-socioeconomic status interaction (GSI) model

In the initial stage of our analysis, we compared the performance of the genotype-by-socioeconomic status interaction (GSI) model to that of the polygenic model. We showed a significant improvement of the GSI model over the polygenic model only when EDU, and not INC or SEI, served as the environmental index Table 2.

**TABLE 2:** Polygenic model vs. full genotype-by-socioeconomic status interaction (GSI) model.

Trait	Environment	Models	Ln likelihood	Likelihood ratio test	*p*-value
FRS-08	EDU	Polygenic	−547.8467	10.303	0.01
		GSI	−542.6951		
FRS-08	INC	Polygenic	−470.1946	3.2985	0.27
		GSI	−468.5453		
FRS-08	SEI	Polygenic	−531.3359	0.5744	0.83
		GSI	−531.0487		
PCE-AA	EDU	Polygenic	−544.9318	20.6511	7.9E-05
		GSI	−534.6063		
PCE-AA	INC	Polygenic	−475.9489	5.1319	0.11
		GSI	−473.3829		
PCE-AA	SEI	Polygenic	−535.4172	0.9728	0.71
		GSI	−534.9308		
PCE-CA	EDU	Polygenic	−558.9761	9.3361	0.02
		GSI	−554.3081		
PCE-CA	INC	Polygenic	−493.6883	1.5337	0.46
		GSI	−492.9215		
PCE-CA	SEI	Polygenic	−549.9187	1.7724	0.52
		GSI	−549.0324		
CCA-IMT	EDU	Polygenic	−272.8105	22.7900	2.8E-05
		GSI	−261.4156		
CCA-IMT	INC	Polygenic	−261.2915	4.3797	0.17
		GSI	−259.1017		
CCA-IMT	SEI	Polygenic	−269.7903	2.1078	0.45
		GSI	−268.7363		
CCA-FIMT	EDU	Polygenic	−276.8565	8.9098	0.02
		GSI	−272.4017		
CCA-FIMT	INC	Polygenic	−258.6960	4.7978	0.14
		GSI	−256.2971		
CCA-FIMT	SEI	Polygenic	−270.5050	2.1992	0.43
		GSI	−269.4054		
ICA-IMT	EDU	Polygenic	−265.9061	23.4829	2.0E-05
		GSI	−254.1646		
ICA-IMT	INC	Polygenic	−256.1566	0.6870	0.79
		GSI	−255.8131		
ICA-IMT	SEI	Polygenic	−261.3151	1.0533	0.69
		GSI	−260.7885		
ICA-FIMT	EDU	Polygenic	−254.5134	35.1676	6.8E-08
		GSI	−236.9297		
ICA-FIMT	INC	Polygenic	−243.4058	0.3634	0.89
		GSI	−243.2240		
ICA-FIMT	SEI	Polygenic	−249.0360	0.5691	0.83
		GSI	−248.7515		

Further Analysis: Additive Genetic Variance Homogeneity, Genetic Correlation, and Residual Environmental variance homogeneity, specifically for the genotype-by-EDU interaction (GEdI) models.

Moving to the second stage, we focused on more specific hypotheses related to additive genetic variance homogeneity, a genetic correlation of 1, and residual environmental variance homogeneity, specifically for the genotype-by-EDU interaction (GEdI) model.

### FRS-08

**FIGURE 1:**
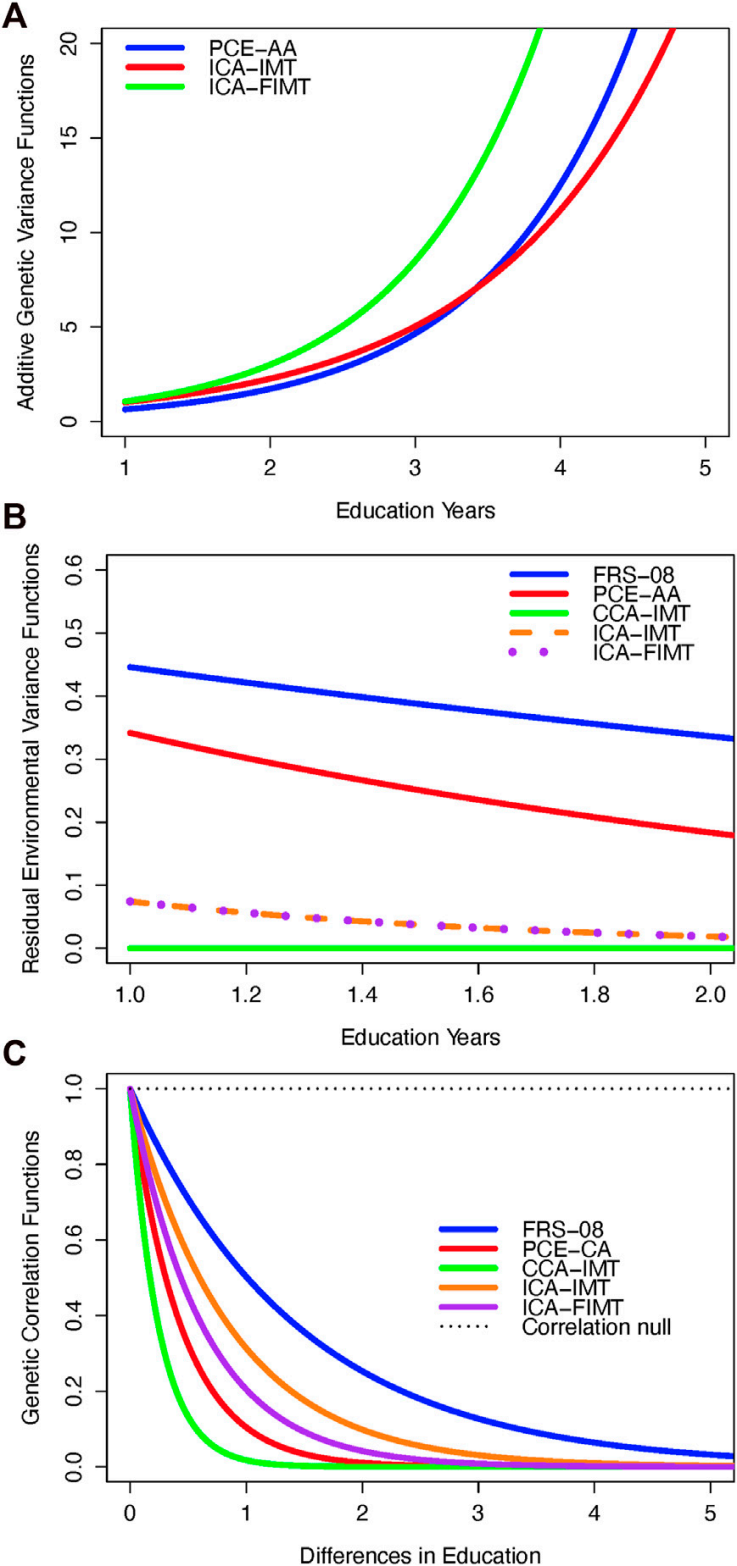
Variance and correlation functions under the genotype-by-education interaction model. **(A)** Additive genetic variance functions. **(B)** Residual environmental variance functions. **(C)** Genetic correlation functions.

**FIGURE 2:**
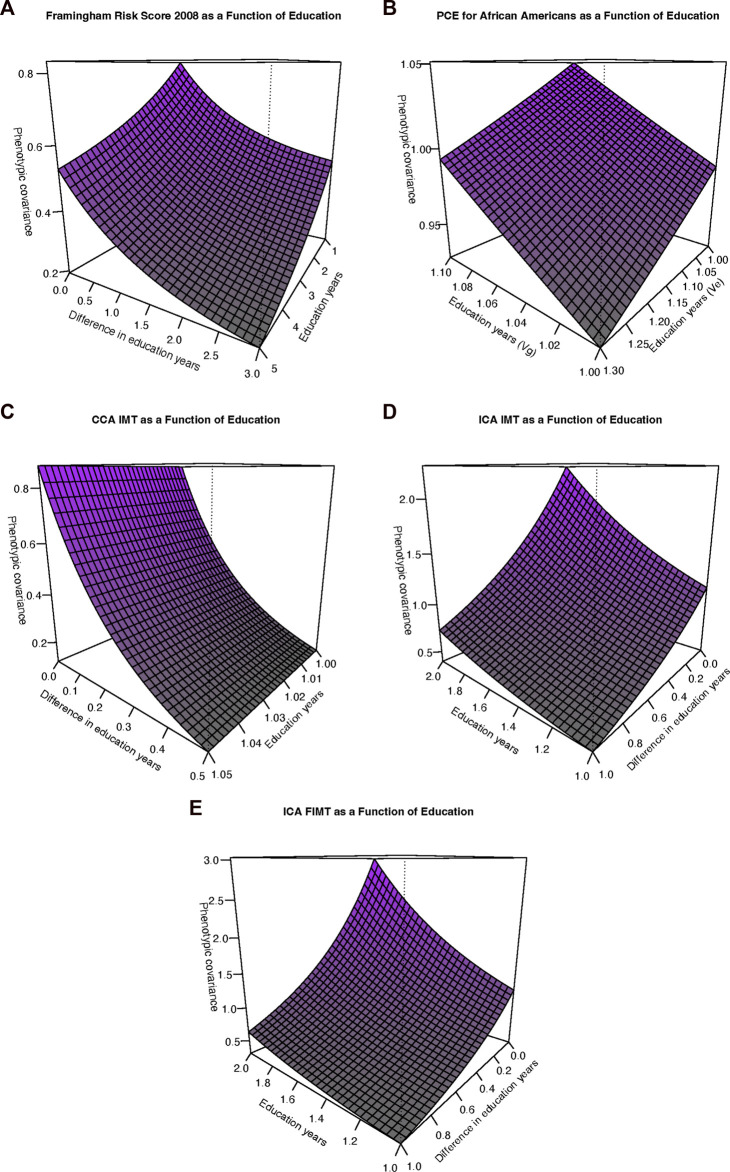
Phenotypic covariance functions. **(A)** Framingham risk score, 2008. The sum of the additive genetic and residual environmental variances increases along the “Education years” (Edu) axis whereas the genetic correlation function decreases away from 1 with increasing differences along the “Difference in education years” (Diff) axis. **(B)** Pooled cohort equations (PCE) for African Americans (PCE-AA). The additive genetic variance (Vg) increases for increasing values along the Edu axis whereas the residual environmental variance decreases for increasing Edu axis values. **(C)** Common carotid artery (CCA) Intima-media thickness (IMT). Axes as in **(A)**. However, as also depicted in Figure 2B, the residual environmental variance is in fact decreasing but at a relatively slower rate (but still statistically significant). **(D)** Internal carotid artery (ICA) IMT. Axes as in **(A)**. The phenotypic covariance is increasing overall as a function of increasing additive genetic variance, decreasing residual environmental variance, and a genetic correlation significantly declining away from 1. **(E)** ICA FIMT. Axes as in **(A)**. Interpretation as in **(D)**.

For FRS-08, we found that the GSE (genetic correlation) was not significantly different from 1. Under a reduced GEdI model (where the gamma-G parameter was always constrained to 0), we discovered significant evidence of GEdI. This was indicated by a genetic correlation function that declined significantly away from 1 (Table 3; Figures 1C, 2A), as well as significant heterogeneity in the residual environmental variance (Table 3; Figures 1B, 2A). Notably, the residual environmental variance decreased as EDU values increased. All other traits were analyzed under the full GEdI model because the parameter standard errors were always less than their MLEs.

**TABLE 3:** Genotype-by-education interaction (GEdI) effects on heart disease risk variables^a^.

Trait	Function	Model (null vs. alternative)^c^	Ln likelihood	Likelihood ratio test	*p*-value
FRS-08^b^	Genetic correlation	Null: $\lambda_{g}=0$	−544.5313	3.2609	0.0355
		Alt: $\tilde{\theta }$	−542.9009		
	Environmental variance	Null: $\gamma_{e}=0$	−546.3321	6.8624	0.0088
		Alt: $\tilde{\theta }$	−542.9009		
PCE-AA	Genetic variance	Null: $\gamma_{g}=0$	−538.4800	7.7474	0.0054
		Alt: $\hat{\theta }$	−534.6063		
	Genetic correlation	Null: $\lambda_{g}=0$	−535.8287	2.4448	0.0590
		Alt: $\hat{\theta }$	−534.6063		
	Environmental variance	Null: $\gamma_{e}=0$	−539.1379	9.0632	0.0026
		Alt: $\hat{\theta }$	−534.6063		
PCE-CA	Genetic variance	Null: $\gamma_{g}=0$	−554.3091	0.0021	0.9633
		Alt: $\hat{\theta }$	−554.3081		
	Genetic correlation	Null: $\lambda_{g}=0$	−557.7770	6.9378	0.0042
		Alt: $\hat{\theta }$	−554.3081		
	Environmental variance	Null: $\gamma_{e}=0$	−554.5175	0.4188	0.5175
		Alt: $\hat{\theta }$	−554.3081		
CCA-IMT	Genetic variance	Null: $\gamma_{g}=0$	−261.5265	0.2219	0.6376
		Alt: $\hat{\theta }$	−261.4156		
	Genetic correlation	Null: $\lambda_{g}=0$	−270.2954	17.7596	1.3E-05
		Alt: $\hat{\theta }$	−261.4156		
	Environmental variance	Null: $\gamma_{e}=0$	−264.1498	5.4686	0.0194
		Alt: $\hat{\theta }$	−261.4156		
CCA-FIMT	Genetic variance	Null: $\gamma_{g}=0$	−274.2249	3.6466	0.0562
		Alt: $\hat{\theta }$	−272.4017		
	Genetic correlation	Null: $\lambda_{g}=0$	−273.5546	2.3059	0.0644
		Alt: $\hat{\theta }$	−272.4017		
	Environmental variance	Null: $\gamma_{e}=0$	−274.1815	3.5597	0.0592
		Alt: $\hat{\theta }$	−272.4017		
ICA-IMT	Genetic variance	Null: $\gamma_{g}=0$	−257.9995	7.6697	0.0056
		Alt: $\hat{\theta }$	−254.1646		
	Genetic correlation	Null: $\lambda_{g}=0$	−256.3332	4.3370	0.0186
		Alt: $\hat{\theta }$	−254.1646		
	Environmental variance	Null: $\gamma_{e}=0$	−262.0594	15.7894	7.1E-05
		Alt: $\hat{\theta }$	−254.1646		
ICA-FIMT	Genetic variance	Null: $\gamma_{g}=0$	−243.8484	13.8375	0.0002
		Alt: $\hat{\theta }$	−236.9297		
	Genetic correlation	Null: $\lambda_{g}=0$	−240.0164	6.17357	0.0065
		Alt: $\hat{\theta }$	−236.9297		
	Environmental variance	Null: $\gamma_{e}=0$	−248.9148	23.9702	9.8E-07
		Alt: $\hat{\theta }$	−236.9297		

^a^ Test for the residual environmental variance function are included here.

^b^ This is only trait-environment dyad where it was reasonable to exclude a parameter, in this case the gammaG parameter because its SE, was greater than its MLE, and the formal test showed it to be non-significant (see text).

^c^ The alternative (Alt) models are specified either by the parameter vector of the reduced GEdI model, given as $\tilde{\theta }={\left[\alpha_{g},\gamma_{g}=0,\lambda_{g},\alpha_{e},\gamma_{e}\right]}^{\prime }$ , or by the parameter vector of the full GEdI model, given as $\hat{\theta }={\left[\alpha_{g},\gamma_{g},\lambda_{g},\alpha_{e},\gamma_{e}\right]}^{\prime }$ . The null models have the same parameter configuration but with the tested parameter constrained to 0, as indicated.

### PCE-AA

In the case of PCE-AA, we found evidence of GEdI through significant heterogeneity in additive genetic variance (Table 3; Figures 1A, 2B), which increased with higher levels of EDU. Additionally, we observed a significant decrease in the residual environmental variance as EDU values increased (Table 3; Figures 1A, 2B). It is important to note that whenever there was evidence of significant additive genetic variance heterogeneity for all traits, it was consistently increasing. Similarly, significant residual environmental variance heterogeneity was consistently decreasing. These two functions were independent under our model and free to increase or decrease. This is an important point because the observed pattern is not an artifact or constraint of the model.

### PCE-CA

For PCE-CA., we identified significant GEdI due to a genetic correlation function declining away from 1 with increasing differences in the EDU index (Table 3; Figure 1C).

### CCA-IMT

Our analysis of CCA-IMT revealed evidence of GEdI, as indicated by a genetic correlation function declining away from 1 and significant heterogeneity in the residual environmental variance (decreasing) (Table 3; Figures 1B, C, 2C).

### CCA-FIMT

There was no evidence of GEdI or residual environmental variance heterogeneity for CCA-FIMT (Table 3). The *p*-values were slightly above the nominal significance level (*p*•0.05). It is worth noting that this trait had the smallest sample size among the variables examined (Table 1), suggesting a potential lack of statistical power rather than an absolute absence of an effect.

For both ICA-IMT and ICA-FIMT, there was evidence of GEdI for both mechanisms of additive genetic variance heterogeneity and a genetic correlation not equal to 1 and evidence of residual environmental variance heterogeneity (Table 3; Figures 1A–C, 2D,E).

## Discussion

There is evidence of an interaction of genes and obesity related to CVD in Mexican Americans (Mitchell et al., 1996; MacCluer et al., 1999; Diego et al., 2007). Mexican Americans, like Mexicans, are carriers of risk alleles related to chronic disease, especially CVD and Lipid metabolism, and adversely respond to obesogenic Westernized diets (Hazuda et al., 1988; Hazuda et al., 1991). The current report makes several key additional contributions. Firstly, education influences the genotype-phenotype map underlying CVD risk variables. We observed an increase in additive genetic variance with higher levels of education, implying the expression of different sets of genes at different ends of the education spectrum. While social determinants of health are undeniably important for CVD and overall health outcomes, our findings emphasize that of the three considered SES variables education plays a predominant role. For several traits (PCE-AA, ICA-IMT, and ICA-FIMT), increasing educations levels were associated with higher additive genetic variance and reduced residual environmental variance. Additionally, for four traits (FRS-08, CCA-IMT, ICA-IM, and ICA-FIMT), the genetic correlations function deviated from 1, indicating the expression of different sets of genes across varying environmental conditions. It is important to note that a genetic correlation of less than 1 across environmental differences does not imply diminishing genetic effects, rather the dynamic nature of genetic effects in response to varying levels of education, while the influence of residual environmental factors becomes less prominent. Our findings highlight that genetic effects become increasingly crucial with higher levels of education.

The role of social determinants of health, particularly socioeconomic status (SES), in the development of cardiovascular disease in Mexican Americans has also garnered significant attention from various disciplines. (de Mestral and Stringhini, 2017; Tillmann et al., 2017; Schultz et al., 2018; Carter et al., 2019; Rosengren et al., 2019; Yusuf et al., 2020; Powell-Wiley et al., 2022). Rosengren et al. (2019) reported results consistent with ours---at the same time that there was a significant inverse relationship between education levels and CVD outcomes, there was, at best, a weak inverse relationship and, at worst, no relationship between income levels and CVD outcomes. We found consistent evidence of GXE effects for education, but not for household income or SEI. Using a Mendelian randomization approach, Tillmann et al. (2017) reported that education levels mediated increased genetic predisposition to CVD. Carter et al. (2019) demonstrated that while certain factors such as body mass index, systolic blood pressure, and smoking mediated some of the inverse relationships between education and CVD outcomes, a significant portion of the protective effect of education remains unexplained. Our results indicate that a substantial proportion of this unexplained effect may be attributed to genotype-by-education (GEdI) effects. By explicitly modeling GEdI, we account for effects beyond additive genetic effects and capture the effects triggered in response to the educational environment.

Recognizing education to be crucially important in the genesis of the social gradient, Marmot hypothesized that SDoH in general (Marmot, 2003; Marmot, 2006a; Marmot, 2006b) and education in particular (Marmot, 2015a; Marmot, 2015b) might directly translate to greater degrees of empowerment, autonomy, and control. Our findings suggest that the effects of education arise at the genetic level, not primarily at the socioeconomic level, as Marmot hypothesized. The potential of education to modulate genetic factors underlying disease phenotypes may be the foundational factor to consider in the role of SDoH in the genesis of the social gradient. How genes and environment interact at a molecular level remains a mystery, although recent evidence suggests that inflammatory pathways may be involved (Hartiala et al., 2021).

Our study is unique in several ways. Firstly, this is a family-based design of Mexican American families and is useful for combined linkage and association analysis. The increased genetic relationship among relatives allows the implementation of genetic variance decomposition methods (Blangero et al., 2013). Family-based studies can identify rare variants (Laird and Lange, 2006; Benyamin et al., 2009). Our environmental decomposition methods approach allows for isolation of gene and environmental interaction. It is important to note that family-based studies have a smaller sample size compared to population-based studies but serve to provide important information from a group (Mexican Americans) that historically has not been included in genetic studies.

### Limitations

The Framingham Risk Score could overestimate/underestimate risk in US-Hispanic populations as the magnitude of associations between risk factors differs between race/ethnic groups (Gijsberts et al., 2015). The findings are theoretical and may not extrapolate to gene expression and regulation. The statistics provide valuable insight, however, future gene evaluations, such as mRNA sequencing, will provide helpful information on genes and pathways involved. In addition to education, income, and SEI, other SES candidates that offer a better resolution of socioeconomic determinants of health include measures of individual endowments, such as asset and human capital, as well as the measure of external constraint from family and culture assimilation, such as family, community, society, and the systems of governance.

## Implications for research

Since education significantly affects genetic factors underlying CVD risk variables, future studies should prioritize education as the critical socioeconomic determinant when investigating gene-environment interactions for CVD risk. Future studies should investigate how education may directly affect genetic factors underlying CVD risk and the mechanisms through which it may exert its influence. We can also examine the genetic correlation across different education levels---the genetic correlation of CVD risk variables was found to decline with increasing differences in education levels. This suggests different genes may be involved in CVD risk at different education levels. Future studies should further examine this relationship and the specific genetic variants that may be involved. Researchers can consider the interplay between genetic and environmental factors. Our findings suggest that genetic and residual environmental factors significantly influence CVD risk variables, with environmental factors becoming less important as education levels increase. Future research should continue to investigate this complex interplay and how genetic and environmental factors interact to impact CVD risk across varying education levels.

The next step in this research is to determine the genes involved in the genotype by education interaction. Once identified, established canonical pathways and molecular expression can provide intervention targets and a better understanding of how genes interact with environments to modify genetic expression in health and illness.

## Conclusion

We examined potential GxE interaction using likelihood-based statistical inference in the phenotypical expression of CVD. We assessed Social Determinants of Health (SDoH), specifically measurements of socioeconomic level, and identified significant GxE interactions for CVD and education. These findings provide evidence that genetic factors interact with the level of education to influence CVD. Future directions will focus on the specific genes involved and the nature of the interactions.

## Data Availability

The raw data supporting the conclusion of this article will be made available by the authors, without undue reservation.

## Abbreviations

American College of Cardiology/American Heart Association, (ACC/AHA); cardiovascular disease, (CVD); common carotid artery far-wall IMT, (CCAFIMT); common carotid artery IMT, (CCAIMT); Duncan’s socioeconomic index, (SEI); education years, (EDU); Framingham Risk Score, (FRS-08; genotype-by-education, (GEdI); genotype-by-environment interaction, (GxE); genotype-by-SES interaction, (GSI); genotype-by-socioeconomic status interaction, (GSI); household income, (INC); internal carotid artery far-wall IMT, (ICAFIMT); internal carotid artery IMT, (ICAIMT); Mexican American Family Studies, (MAFS); pooled Cohort Equation Risk Score African Americans, (PCE-AA); pooled Cohort Equation Risk Score Caucasian Americans, (PCE-CA); socioeconomic status, (SES).
